# New questions and avenues for research regarding interpretation of the significance of respiratory viruses adrift in the air

**DOI:** 10.1016/j.ebiom.2024.105524

**Published:** 2025-01-02

**Authors:** John A. Lednicky

**Affiliations:** College of Public Health and Health Professions, Department of Environmental and Global Health, University of Florida, USA

Respiratory infections are the leading reason for hospitalisations worldwide.^1^ While numerous human respiratory viruses have been identified, the manner by which they are transmitted person-to-person is less understood than the situation with their bacterial counterparts. The same is true for the respiratory fungus *Pneumocystis jirovecii.* A major reason that knowledge of the transmission dynamics of respiratory viruses has lagged compared to what is known for bacteria such as *Mycobacterium tuberculosis* and for fungi revolves around the difficulty of collecting airborne viruses.^2^ Whereas airborne bacteria and fungi are readily collected from air samples using commonly used air samplers, the smaller size and low mass of virus particles makes their collection technically challenging.^2^ Knowledge gaps regarding respiratory virus transmission were evident during the COVID-19 pandemic, wherein many public health authorities (a) recommended frequent handwashing, and (b) maintenance of one metre distances between people to prevent or at least reduce the chances of being infected by SARS-CoV-2 via droplets. Little attention was paid at the onset of the pandemic to the risks of exposure to airborne SARS-CoV-2, whereas it is now acknowledged that inhalation exposure is the major route the virus spreads person-to-person.

Insights regarding the occurrence of airborne viruses in healthcare settings have accelerated as better instruments for the collection of viruses in aerosols have become available.^3^, ^4^, ^5^, ^6^ Indeed, air-sampling studies revealed that SARS-CoV-2 is released into air by COVID-19 patients,^7^, ^8^, ^9^ and that has led to the development of improved infection control measures in clinical practice and in the design of ventilation systems for indoor spaces in homes, workplaces, and commercial properties.

It is important to note that the detection by PCR or antigen-based technologies of microorganisms collected by air sampling does not prove that they pose an exposure risk. Airborne pathogens are potentially hazardous when inhaled, and some infect humans following their contact deposition onto mucus membranes of the eyes or mouth. This is an important concept, especially with regard to airborne viruses, as the air that animals and humans breathe is full of virus particles. We constantly breathe in viruses that are capable of infecting animals, bacteria, fungi, plants, and humans. But, only the subset of viruses that can cause human illness through inhalation exposure would be of medical concern. Further, many of the viruses we inhale are non-viable (not “live”) due to the detrimental effects of desiccation, UV irradiation, etc., and only viable viruses (“virions”) can cause clinically apparent illnesses.

How then should researchers interpret the results of microbial analyses of air samples? Now that it is well-accepted that respiratory viruses can be transmitted through airborne routes, attention should now be given to measurement of clinical and epidemiological correlates related to detection of airborne viruses. In the January 2025 issue of *eBioMedicine*, Dr. Caspar Geenen et al.^10^ probe the link between respiratory pathogens collected from the air with those detected in nasal mucus deposited on tissues by people in a childcare setting. This approach helps us understand a “bigger picture”. Indeed, the report by Dr. Greenen et al. stands out for three reasons: (a) they performed air samplings in a childcare setting for 22 consecutive weeks, whereas the duration of such sampling studies is much shorter in work performed by others, (b) they tested the material collected by air-samplers using PCR for 29 respiratory pathogens, whereas others generally test for fewer types of microorganisms, and (c) they compared and contrasted the identity of the airborne pathogens with those detected in mucus deposited in tissues by the attendees during the same time period. We learn several important lessons from their work. It will come as a surprise to many that the authors found SARS-CoV-2 more often in air than in mucus samples.

Unlike what was reported early on during the COVID-19 outbreak, these observations are consistent with newer findings, including work performed by myself and collaborators. Not surprisingly, dissimilar results were obtained when different viruses were analysed by Dr. Geenen et al.^10^ For example, human coronavirus NL63 was more frequently detected in nasal mucus than in the air, in contrast to SARS-CoV-2, even though both are coronaviruses. Many epidemiological pathogen transmission studies, including those for airborne viruses, focus on one type of virus, with the results assumed to be representative for all viruses. Finding *P. jirovecii* in the air of a nursery is of interest to the clinic. Little is known about the prevalence of this organism in breathing air. Is this an organism that has been overlooked as a common human pathogen?

The one shortcoming of the study of Geenen et al. is that virus viability was not confirmed. Only virions can induce illness. Confirmation of virus viability, in both virions in mucus and those in the air, would be a good topic for follow-up work.

## Contributors

JAL is the sole author.

## Declaration of interests

The author declares no conflicts of interest.
